# Effect of Rhizome Fragment Length and Burial Depth on the Emergence of a Tropical Invasive Weed *Cyperus aromaticus* (Navua Sedge)

**DOI:** 10.3390/plants11233331

**Published:** 2022-12-01

**Authors:** Aakansha Chadha, Singarayer K. Florentine, Kunjithapatham Dhileepan, Christopher Turville

**Affiliations:** 1 Future Regions Research Centre, Federation University Australia, Mount Helen, VIC 3350, Australia; 2 Applied Chemistry and Environmental Science, School of Science, STEM College, RMIT University, 124 La Trobe St, Melbourne, VIC 3000, Australia; 3 Department of Agriculture and Fisheries, Biosecurity Queensland, Ecosciences Precinct, Dutton Park, QLD 4102, Australia; 4 Institute of Innovation, Science and Sustainability, Federation University Australia, Mount Helen, VIC 3350, Australia

**Keywords:** burial depth, rhizome, soil, regeneration, perennial weed, integrated weed management, sedge, vegetative reproduction, *Kyllinga polyphylla* Willd. ex Kunth, *Kyllinga aromatica* Ridley, Greater Kyllinga

## Abstract

*Cyperus aromaticus* (Navua sedge) is a problematic perennial weed in pastures and crops including sugarcane, banana, rice, and fruits and vegetables in tropical climates. It reproduces both via rhizomes and seeds. As a regenerative and storage organ, these rhizomes play an important part in the invasion, establishment, and persistence of this weed. To eliminate their regenerative ability, it is important to understand the regrowth potential with respect to rhizome fragment size and burial depth. This study evaluated the emergence of *C. aromaticus* from rhizomes in a controlled condition. Three different sizes of rhizome fragments were buried at seven depths of up to 20 cm in two soil types. The experimental measurements included (i) the time for tillers to emerge, (ii) the cumulative emergence of tillers, recorded weekly, and (iii) the number of underground emerging tillers. The cumulative shoot emergence and the number of underground tillers produced were found to be positively correlated with the initial length of the rhizome fragments and negatively correlated with the burial depth. The time for the emergence of the tillers was negatively correlated with the burial depth, and soil type had no significant effect on any of the parameters recorded. There was no emergence recorded from rhizomes buried at 15 cm depth and deeper, irrespective of their size. Our results indicate that the combination of the fragmentation of rhizomes into small pieces and a deep burial, below 15 cm, is an important aspect to control the regeneration of *C. aromaticus* from rhizomes, if tillage is carried out, and can therefore form a part of an integrated weed management strategy for this troublesome weed.

## 1. Introduction

*Cyperus aromaticus* (Ridley) Mattf. & Kükenth (Navua sedge) is an invasive C_4_ perennial sedge species, which is found predominantly in tropical environments. It is an aggressive weed that causes significant impacts on livestock grazing industries and sugarcane and banana plantations and also affects a range of native ecosystems in tropical north Queensland in Australia [1]. It can quickly overrun pastures, and because it is unpalatable, it provides little feed value for cattle [2]. If pastures are overgrazed, this sedge can rapidly become permanently established and eventually dominate the area. Coupled with this invasion, *C. aromaticus* is capable of spreading at an alarming rate across larger areas within a short period of time [3]. A significant contributor to the spread of this species is its dual mode of reproduction, via seeds and vegetatively by the extension and fragmentation of the underground rhizome system [4]. Rhizomes and rhizome fragments resulting from disintegration of the parent plant retain their reproductive function within the dispersed units and may lead to the establishment of a bud bank in addition to the seed bank [5]. The *C. aromaticus* plant persists by means of a superficially placed sympodial rhizome system primarily found in the top 5–7 cm of the soil surface (A. Chadha, unpublished). The rhizome is formed by many conical basal swellings joined together at their bases, and from the apex of the conical swelling, the aerial shoot arises. The basal swelling consists of several closely situated nodes (usually 1–5), enclosed by scale leaves [3]. Some of these scale leaves have buds in their axils. There is no bud present in the first node. The axillary bud present in the second node gives rise to new tillers. A large, well-developed potential bud, which is normally dormant, is present in the third node and is responsible for branching by giving rise to a new shoot [3]. A small dormant bud is present in the fourth node, which does not sprout. The rhizome chain characteristically radiates outwards as new shoots are added on during its dynamic growth and development.

This dual mode of reproduction implies that the management of this species should target control of both the aboveground and underground systems rather than the aboveground biomass alone [6]. Studies conducted on *C. aromaticus* show that sequential applications of herbicide are required for the control of this species [2,7], and currently only one herbicide, halosulfuron-methyl (Sempra), is registered for its control in pastures. However, a single application of halosulfuron-methyl affects the aboveground foliage, but does not kill the rhizome [8]. In addition, relying on and using one herbicide continuously will seriously increase the chance of herbicide resistance [9,10]. Hence, to better manage *C. aromaticus* in an ecologically sustainable way, simultaneously targeting both the above- and belowground biomasses without the excessive use of herbicides is essential.

Rhizomes serve two-fold functions as organs for survival and propagation; they are the main reserves of carbohydrate as well as the housing of the dormant buds for survival during unfavourable periods [11,12]. The production of vegetative buds provides plants with a safety net for regrowth or reproduction if harsh environmental conditions result in the death of actively growing or metabolising tissues [13]. These vegetative buds contain meristems in several stages of growth and development, which possess the capacity to serve as reservoirs for potential vegetative development [13,14]. However, not all the vegetative buds grow at the same time, with some being dormant due to restrictions in bud activity, which is one of the significant challenges to deal with while managing weeds with underground rhizomes [15]. Another reason for the persistence of rhizomes is their ability to store nutrient reserves, which enables them to withstand long periods of dormancy and to support respiration and formation of new shoots and roots in the early autotrophic stages [16]. The total quantity of non-structural carbohydrates present in the rhizome is important for the growth of new organs and is highly correlated with the resprouting and regrowth potential of the plant and aboveground phenological development [17,18]. Thus, targeting the rhizomes, either by killing them or stopping regrowth from them, is a critical step in the management of *C. aromaticus*.

Mechanical intervention forms an important part of the integrated weed management system to reduce the reliance on herbicides [19]. In this regard, soil cultivation and tillage could be used as mechanical methods for weed control, which would result in either fragmentation of the rhizomes, burial of the rhizomes, or both fragmentation and burial together. Typically, cultivation mechanically breaks the rhizomes into small fragments, which would ideally break apical dormancy and release a proportion of dormant buds to develop into shoots, thus increasing the spread [20,21]. However, upon cultivation, shoots develop from vegetative fragments, and, in the process, bud sprouting, and the growth of emerged shoots consume a high proportion of stored energy in the rhizomes [22,23].

Regeneration from buried rhizomes and their fragments is an important phenomenon contributing to the rapid increases in population of rhizomatous weeds, such as *C. aromaticus*, making understanding the effect of burial depth on the regeneration from rhizomes an important control issue [24]. Studies conducted on other rhizomatous weed species suggest that shoot emergence from a rhizome fragment is strongly dependent on the rhizome size and the burial depth of the rhizome [25,26,27,28,29]. Typically, it is expected that deep burial of short rhizome fragments will reduce shoot emergence. Apart from these two elements, an external factor, soil type, can also influence the emergence of shoots from the rhizomes [30]. The purpose of this study was to investigate the effect of rhizome fragment lengths and burial depths on the emergence of *C. aromaticus* to gain knowledge about fragmentation and establishment dynamics under various soil types. To help to systematise our study, we hypothesised that:

**Hypothesis** **(i).** *Total emergence of tillers is positively correlated with length of rhizome fragment and negatively correlated with burial depth.*

**Hypothesis** **(ii).** *Time to emergence of tillers is positively correlated with length of rhizome fragment and negatively correlated with burial depth.*

**Hypothesis** **(iii).** *Total emergence and time to emergence are dependent on soil type.*

## 2. Results

The results obtained from the mixed models for the cumulative emergence of tillers are summarised in Table 1. Soil did not have a significant effect on the emergence of tillers; however, burial depth, rhizome size, and their interaction all had significant effects (Table 1). In addition, time, interaction between burial depth and time, and interaction between rhizome size and time had significant effects on the emergence of tillers (Table 1).

**Figure 1:**
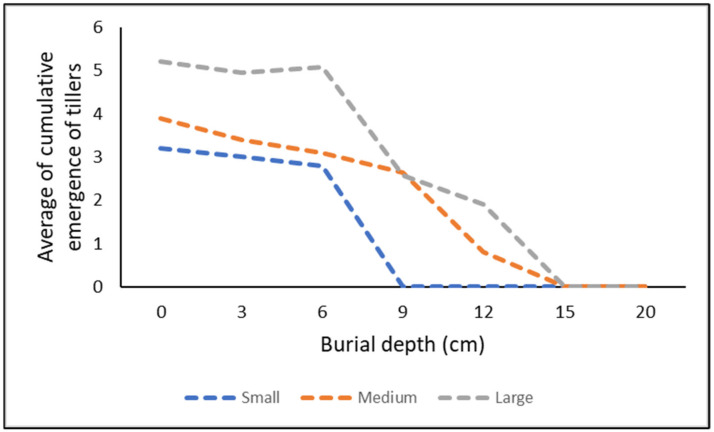
Average of cumulative emergence of tillers from buried *Cyperus aromaticus* rhizome fragments of different sizes over a period of 70 days. Emergence was averaged for both the soil types used.

**Figure 2:**
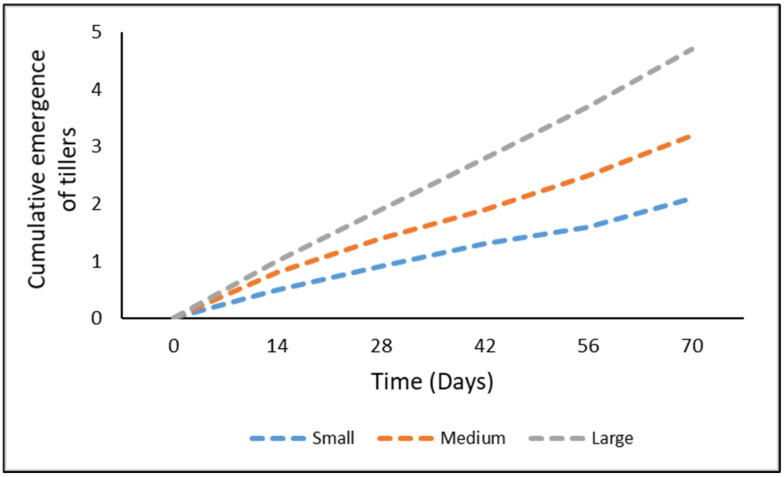
Cumulative emergence from buried *Cyperus aromaticus* rhizome fragments of different sizes over a period of 70 days. Emergence was averaged for all the burial depths and both the soil types used.

**Figure 3:**
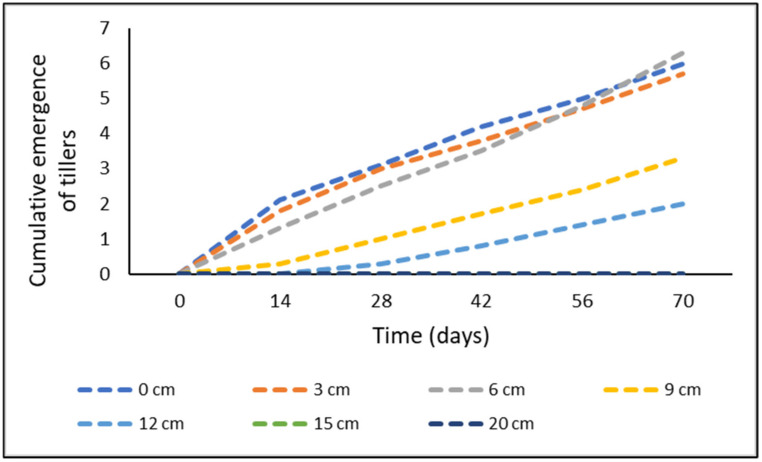
Cumulative emergence from *Cyperus aromaticus* rhizome fragments buried at various depths over a period of 70 days. Emergence was averaged for all the rhizome sizes and both soil types used.

Overall, the statistical analyses support the hypothesis (i) that total emergence is positively correlated with the length of the rhizome fragment and negatively correlated with the burial depth (Figure 1). An examination of the interaction effect indicated that at days 14 and 28, there was no significant difference (*p* ≥ 0.05) in the emergence from any of the rhizome sizes (Figure 2). However, at days 42, 56, and 70, the emergence from the large rhizome was significantly higher than from the other two rhizome sizes (*p* < 0.05) (Figure 2). At day 70, the highest emergence of tillers was observed in the large rhizome fragment, followed by medium-sized fragments, and the least amount of emergence was recorded in the small rhizomes (Figure 2). A decrease in emergence was observed as the burial depth increased (Figure 3). The emergence from burial depths 0, 3, and 6 cm was similar (*p* ≥ 0.05) throughout the experiment, and these depths were significantly higher than the emergence from 9 and 12 cm burial depths (*p* < 0.05) (Figure 3). There was no emergence from any of the rhizomes buried at depths 15 cm and beyond (Figure 3).

Hypothesis (ii) stating that the time to emergence of the tillers is positively correlated with the length of the rhizome fragment and negatively correlated with the burial depth was partially supported by the statistical analyses. There was a significant negative relationship between the burial depth and the time to emergence (*p* < 0.001). However, there was no significant effect of the rhizome size on the time to emergence (*p* = 0.943) (Table 2). For the two soil types studied in this experiment, hypothesis (iii) stating that the total emergence and time of emergence are dependent on soil type was not supported. Neither the total emergence of tillers (*p* = 0.622), nor the time to emergence (*p* = 0.529) was influenced by the soil types (Table 1 and Table 2).

The burial depth and rhizome size individually had significant effects on the number of underground emerging tillers (*p* < 0.001) (Table 2). The number of underground emerging tillers was significantly higher in the large rhizomes compared to the small and medium rhizomes (*p* < 0.05). Underground emerging tillers from rhizomes buried at 6, 9, and 12 cm depth were similar in number and significantly higher than those buried at 15 and 20 cm depths. None of the buried rhizomes died.

## 3. Discussion

The emergence from the rhizome decreased as the burial depth of the rhizome increased. A decline in emergence with increasing burial depth has also been observed in other rhizomatous species, such as *Tussilago farfara* (Coltsfoot), *Physalis viscosa* (Prairie groundcherry), and *Cynodon dactylon* (Bermudagrass) [26,27,31]. The energy required by the emerging tillers to reach the soil surface increases with the increase in burial depth, and the regrowth from the rhizomes may fail to reach the surface due to insufficient nutrient reserves [29]. One of the primary reasons for the persistence of rhizomatous weeds, such as *C. aromaticus,* is their ability to store a large amount of food reserves in their rhizomes [32,33,34]. The total of non-structural carbohydrates contained in the rhizomes is highly correlated with the resprouting and regrowth potential of the plant and aboveground phenological development [18].

The hypothesis that the total emergence of tillers from the rhizomes is positively correlated with the rhizome size holds true in the case of *C. aromaticus*. At all the burial depths, where emergence was recorded, the total emergence of tillers from small rhizomes was lower than those emerging from large rhizomes. With increasing burial depth, the effects of the rhizome size became stronger. An increase in burial depth is more detrimental to the survival of shorter rhizome fragments compared to larger fragments due to a greater chance of survival of buds on larger rhizomes as there are more buds, whilst bigger rhizomes also have more energy reserves to help tillers emerge from greater burial depths [35]. Sprouting from rhizome fragments and regrowth consumes the stored energy in the rhizomes, so the more the rhizomes are fragmented, the more energy is used in regeneration [22].

In the case of mechanical control, the number of tillers emerging from a rhizome following cultivation is determined by both the lengths to which the rhizomes are broken and the depth to which fragments are buried. Logically, fragmentation by itself will increase the potential for rhizomatous weed spread due to breaking of apical dormancy, but the process of fragmentation can also lead to deep burial of rhizome pieces [25]. Thus, as a management approach, tillage has a dual advantage [31]. The first advantage is that deeply buried and smaller fragments lead to emerging plants with less stored energy. The deeper the burial and the smaller the rhizome fragment will mean more energy will be used for shoot emergence [11]. The second advantage is that ungerminated buds will have lower reserves in the rhizome fragment for shoot emergence [26].

The hypothesis that the time to emergence was negatively correlated with the burial depth was supported in this study. The deeper the fragments of *C. aromaticus* were buried, the longer it took them to emerge. This delay in weed emergence time allows the competing crop to establish in the field thus weakening the weed’s ability to compete [28]. In addition, the later a shoot emerges, the less time is available in the growing season, thus producing less biomass and accruing less nutrients for storage in the rhizome to drive growth in the next season [31].

As we determined that none of the rhizomes died in the experiment, the observation that none of the underground emerging tillers present had emerged from the soil, leaves the possibility open that they could have emerged if the experiment continued longer. Hence, it is recommended that future studies of this type continue for a longer duration to check whether the developing underground tillers would eventually emerge, which has implications for the fate of the rhizomes.

In conclusion, there was no emergence recorded from *C. aromaticus* rhizomes buried at 15 cm depth and deeper, irrespective of their size in this study. Our results indicate that the combination of fragmentation of rhizomes into small pieces and deep burial, below 15 cm, is an important aspect to control the regeneration of *C. aromaticus* from rhizomes, if tillage is carried out, and can therefore form a part of an integrated weed management strategy for this troublesome weed. This study on the propagation/sprouting of *C. aromaticus* from rhizomes of different sizes and at different depths is important in devising integrated management strategies. The information provided here can be applied to other invasive species with vegetative and sexual reproduction, thus helping to control other species.

## 4. Materials and Methods

### 4.1. Collection and Classification of Plant Material

The experiment was conducted between April and July 2020 at the Mount Helen campus of Federation University, Australia (37°37′41.4″ S, 143°53′26.4″ E). Rhizomes of *C. aromaticus* were collected from the banks of Nyleeta creek (17°47′27.204″ S, 145°57′18.18″ E), near Innisfail, Far North Queensland in April 2020. This is a rainforest creek, upstream from most agricultural and other soil disturbance. The rhizomes were dug up from the sandy substrate of the creek banks. The aboveground parts were removed, and then the rhizomes were washed and wrapped in moist paper towels to keep them moist until they were potted in the glasshouse four days later. Size classes were chosen in order to encompass the variation in length observed among the collected rhizomes. The study sample contained 168 rhizomes, which were classified into three length categories: (i) small fragment (3–5 cm in length, 4.2 cm mean ± 0.10 SE); (ii) medium fragments (5.1–8 cm in length, 6.7 cm mean ± 0.14 cm SE); and (iii) large fragments (8.1–10 cm in length, 9.3 cm mean ± 0.11 cm SE).

### 4.2. Experimental Set Up

The experiment was conducted on a completely randomised design, using a three-factorial pot approach, where the factors were (i) soil type, (ii) rhizome length, and (iii) burial depth. Four replicates were carried out for each treatment, leading to 2 soil types × 7 depths × 3 rhizome lengths × 4 replicates = 168 pots. The factors were chosen to understand the response of *C. aromaticus* rhizomes to soil types, burial depths, and fragment sizes and to expand our knowledge of the conditions affecting the emergence of tillers from rhizomes. The pots were placed randomly in the glasshouse, maintained at day temperatures between 32 °C and 27 °C and a night temperature between 23 °C and 18 °C, and kept at a relative humidity above 80%. The pots were sprinkler-irrigated using an automatic watering system for 10 min daily to avoid any moisture stress.

Two types of soils were used for the experiment: (i) clay loam (32% clay, 64% silt, and 4% sand) and (ii) sandy loam (55% sand, 35% silt, and 10% clay). For each of the soil types, rhizomes were buried at seven different depths; 0, 3, 6, 9, 12, 15, and 20 cm. Cylindrical pots measuring 32 cm height and 15 cm diameter were partially filled with the relevant soil; the rhizome was placed on to the surface, and additional soil was added to create the required burial depth. Soil was packed before and after placing the rhizome by tapping on to the bench. Each of the soil type and burial depth variations had three sizes of rhizomes; small, medium, and large as the third factor.

**Figure 4:**
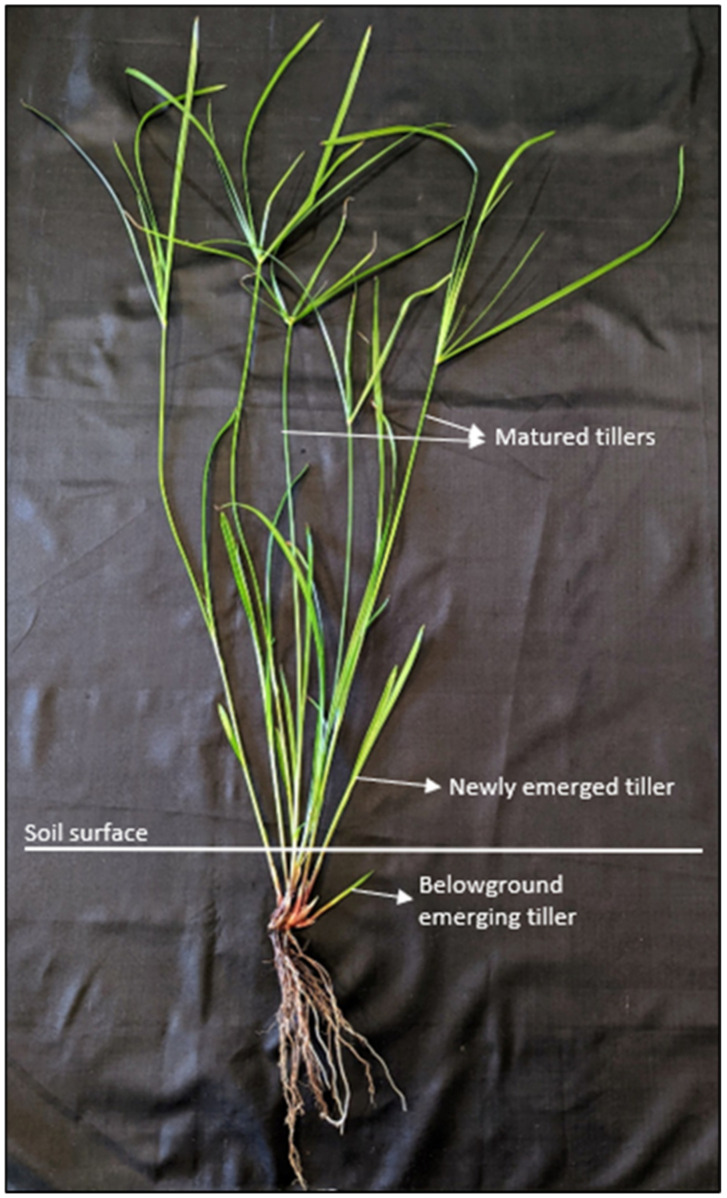
Classification of underground emerging tillers, newly emerged tillers. and matured tillers of *Cyperus aromaticus*.

Cumulative emergence of tillers (including newly emerged tillers and matured tillers) was recorded every seven days from the commencement of the experiment until day 70. At the end of the experiment, rhizomes were carefully removed; their status was checked and recorded as ‘virtually healthy’ or ‘decayed’, and the number of underground emerging tillers was counted. Figure 4 shows the classification of underground emerging tillers, newly emerged tillers, and matured tillers of *C. aromaticus*.

### 4.3. Statistical Analyses

Linear mixed models were conducted using SPSS to investigate the main effects of soil, burial depth, rhizome size, time, and their 2-way interactions on the cumulative emergence of tillers. Time was treated as a random effect to account for the emergence from the same rhizome being measured on several occasions. Time to emergence and the number of underground tillers were analysed using general linear models with soil, burial depth, and rhizome size as the main effect and their 2-way interactions. The significance of the main effects was analysed using Tukey’s post hoc analysis, and significant interactions from the mixed models were analysed by investigating the simple main effects with Bonferroni adjustments. All assumptions were checked by investigating the normality and spread of the residuals.

## Figures and Tables

**Table 1:** Summary of ANOVA for all main effects and their interaction from the mixed models for the cumulative emergence of tillers.

	Cumulative Emergence of Tillers			
	df1	df2	F	*p*-Value
Soil	1	150.937	0.244	0.622
Burial depth	6	150.568	130.481	<0.001
Rhizome size	2	30.318	7.060	0.003
Soil*burial depth	6	11.360	2.469	0.090
Soil*rhizome size	2	11.414	0.590	0.571
Burial depth*rhizome size	12	11.388	3.317	0.026
Time	4	32.138	126.279	<0.001
Soil*time	4	32.138	0.816	0.525
Burial depth*time	24	32.138	11.281	<0.001
Rhizome size*time	8	32.138	8.525	<0.001

Note: df1, df2, F, and *p*-value refer to the numerator degrees of freedom, denominator degrees of freedom, test statistic, and *p*-value, respectively, for each treatment or interaction effect from the linear mixed model.

**Table 2:** Summary of ANOVA for all main effects and their interactions for the time of emergence of tillers and the underground emerging tillers of *Cyperus aromaticus*.

	Time to Emergence				Underground Emerging Tillers			
	df1	df2	F	*p*-Value	df1	df2	F	*p*-Value
Soil	1	72	0.400	0.529	1	108	0.393	0.532
Burial depth	4	72	120.14	<0.001	5	108	9.718	<0.001
Rhizome size	2	72	0.058	0.943	2	108	14.557	<0.001
Soil*burial depth	4	72	1.546	0.198	5	108	0.689	0.633
Soil*rhizome size	2	72	1.512	0.227	2	108	0.098	0.906
Burial depth*rhizome size	6	72	0.872	0.520	10	108	1.220	0.287

Note: df1, df2, F, and *p*-value refer to the numerator degrees of freedom, denominator degrees of freedom, test statistic, and *p*-value, respectively, for each treatment or interaction effect from the linear mixed model.

## Data Availability

Not applicable.
